# Adaptation and convergence in circadian‐related genes in Iberian freshwater fish

**DOI:** 10.1186/s12862-021-01767-z

**Published:** 2021-03-08

**Authors:** João M. Moreno, Tiago F. Jesus, Maria M. Coelho, Vitor C. Sousa

**Affiliations:** cE3c – Centre for Ecology, Evolution and Environmental Changes, Faculdade de Ciências, Universidade de Lisboa, Campo Grande, 1749-016 Lisboa, Portugal

**Keywords:** Adaptation, Circadian rhythm, Evolutionary convergence, Freshwater fish, Selection

## Abstract

**Background:**

The circadian clock is a biological timing system that improves the ability of organisms to deal with environmental fluctuations. At the molecular level it consists of a network of transcription-translation feedback loops, involving genes that activate (*bmal* and *clock* – positive loop) and repress expression (cryptochrome (*cry*) and period (*per*) – negative loop). This is regulated by daily alternations of light but can also be affected by temperature. Fish, as ectothermic, depend on the environmental temperature and thus are good models to study its integration within the circadian system. Here, we studied the molecular evolution of circadian genes in four *Squalius* freshwater fish species, distributed across Western Iberian rivers affected by two climatic types with different environmental conditions (e.g., light and temperature). *S. carolitertii* and *S. pyrenaicus* inhabit the colder northern region under Atlantic climate type, while *S. torgalensis*, *S. aradensis* and some populations of *S. pyrenaicus* inhabit the warmer southern region affected by summer droughts, under Mediterranean climate type.

**Results:**

We identified 16 circadian-core genes in the *Squalius* species using a comparative transcriptomics approach. We detected evidence of positive selection in 12 of these genes using methods based on dN/dS. Positive selection was mainly found in *cry* and *per* genes of the negative loop, with 55 putatively adaptive substitutions, 16 located on protein domains. Evidence for positive selection is predominant in southern populations affected by the Mediterranean climate type. By predicting protein features we found that changes at sites under positive selection can impact protein thermostability by changing their aliphatic index and isoelectric point. Additionally, in nine genes, the phylogenetic clustering of species that belong to different clades but inhabit southern basins with similar environmental conditions indicated evolutionary convergence. We found evidence for increased nonsynonymous substitution rate in convergent lineages, likely due to positive selection at 27 sites, mostly in *cry* genes.

**Conclusions:**

Our results support that temperature may be a selective pressure driving the evolution of genes involved in the circadian system. By integrating sequence-based functional protein prediction with dN/dS-based methods to detect selection we uncovered adaptive convergence in the southern populations, probably related to their similar thermal conditions.

## Background

Organisms are exposed to daily environmental fluctuations in their natural habitats. To overcome them, organisms developed biological timing systems to optimize their physiological and biochemical processes in space and time [1]. These systems work as internal clocks and require a proper synchronization with environmental signals. Thus, understanding the molecular evolution of the genes involved in the circadian system provide important clues to elucidate how species adapt to their environments.

The circadian system is a universal biological timing system found virtually in all organisms [2]. Circadian system is synchronised by light–dark cycle of a day’s period and present oscillations with a period of ~ 24 h called circadian rhythms. Oscillations are generated and regulated at molecular level, but the outcomes have been shown to influence several aspects of physiology, behaviour and ecology of organisms [2, 3]. In fact, circadian rhythms have been shown to improve the inherent ability of several organisms to survive under changing environments, by aiding them to efficiently anticipate periodic events, specifically light changes and climate seasons [2, 3].

The molecular circadian system consists of a network of signalling transduction pathways regulated mainly by interconnected transcription-translation feedback loops (Additional file 1: Fig. S1) [4, 5]. The regulatory loops are sustained by the so-called core circadian genes and proteins, requiring about 24 h to complete a cycle [1, 5]. In vertebrates, several genes have been reported to be responsible for the maintenance and regulation of the circadian system [1]. The core circadian-genes belong to four main gene families: Cryptochromes (CRY), Period (PER), CLOCK, and BMAL [5]. These gene families encompass several characterized genes (*cry, per, bmal* and *clock*) in vertebrates. In fish, several families possess a larger number of circadian paralogs as compared to other vertebrates [6]. For instance, in the cyprinid zebrafish (*Danio rerio*; Order Cypriniform, suborder Cyprinoidei; Schönhuth et al. 2018), several genes have been identified: six *cry* (*cry1aa, cry1ab, cry1ba, cry1bb, cry2, cry3*), four *per* (*per1a, per1b, per2, per3*), three *bmal* (*bmal1a, bmal1b, bmal2*), and three *clock* (*clocka, clockb, clock2/npas2*) [7–10]. Cryptochrome genes encode for a class of flavoproteins that are sensitive to blue light [10], whereas *period* genes encode for proteins that also display a strong but differential light responsiveness [11, 12]. Both *cry* and *per* were found to be key agents in the entrainment of the circadian system, as they constitute the negative elements of the system (i.e. repressors of transcription) [13]. BMAL (Brain and muscle ARNT like) and CLOCK (Circadian locomotor output cycle kaput) families encode for canonical circadian proteins, a highly conserved bHLH (basic-Helix-Loop-Helix)-PAS (Period-Aryl hydrocarbon receptor nuclear translocator- Single minded) transcriptional factors and are the positive elements of the circadian system (i.e. activators of transcription) [1, 5].

Studies in fish allowed to elucidate the different levels of organization of the circadian system. This is because fish are a very diverse group of animals adapted to nearly all aquatic environments and have a larger number of circadian paralogs as compared to the other vertebrates [6]. The higher number of paralogs is not surprising given the whole-genome duplications that characterize the evolution of teleost-fish [14]. Advances in genome sequencing allowed to identify circadian-related genes in several model organisms, including zebrafish. Homology-based methods allowed the identification of circadian genes in other non-model fish species [7–9, 15]. Some studies cover the evolutionary relationships of the core-clock gene families and the mechanisms driving their molecular evolution [7–10, 16], but several key questions remain open, namely on the role of paralogs, which is higher in fish when compared to other vertebrates, and its importance for adaptation of species to different environments [6].

By sequencing the transcriptome of zebrafish exposed to light, two recent studies identified several genes whose expression depends on light, revealing a multi-level regulation of circadian rhythms by light-cycles [17, 18]. Photoreception is particularly interesting in fish as, contrary to most vertebrates that only perceive light through the eyes, fish also have a photosensitive pineal gland, dermal melanophores, and brain photoreceptors [1]. In addition, fish possess independent peripheral photoreceptors and self-sustaining circadian oscillators in every tissue [1, 19].

Circadian rhythms can also be entrained by temperature [20–23]. In mammals it was demonstrated that peripheral cells *in vitro* could sense the change of room temperature as a cue for entrainment of circadian system [20]. In zebrafish, temperature has also an important role in circadian clock [21, 23], and it was proposed that temperature could entrain the phase of the system by driving expression levels of *per3*, and other circadian genes (namely *cry2* and *cry1ba*) via an alternative hypothetical enhancer [21]. In this model, *per1b* (formerly known as *per4*) promoter integrates temperature and light regulatory inputs [21]. In agreement with the hypothesis that temperature affects the circadian system, in a study comparing a transcriptome profiling of two freshwater fish species (*Squalius carolitertii* and *S. torgalensis*) exposed to different temperatures, Jesus et al. [24, 25] found two differentially expressed genes (*cry1aa* and *per1a*) between a control and a thermal stress condition.

**Fig. 1:**
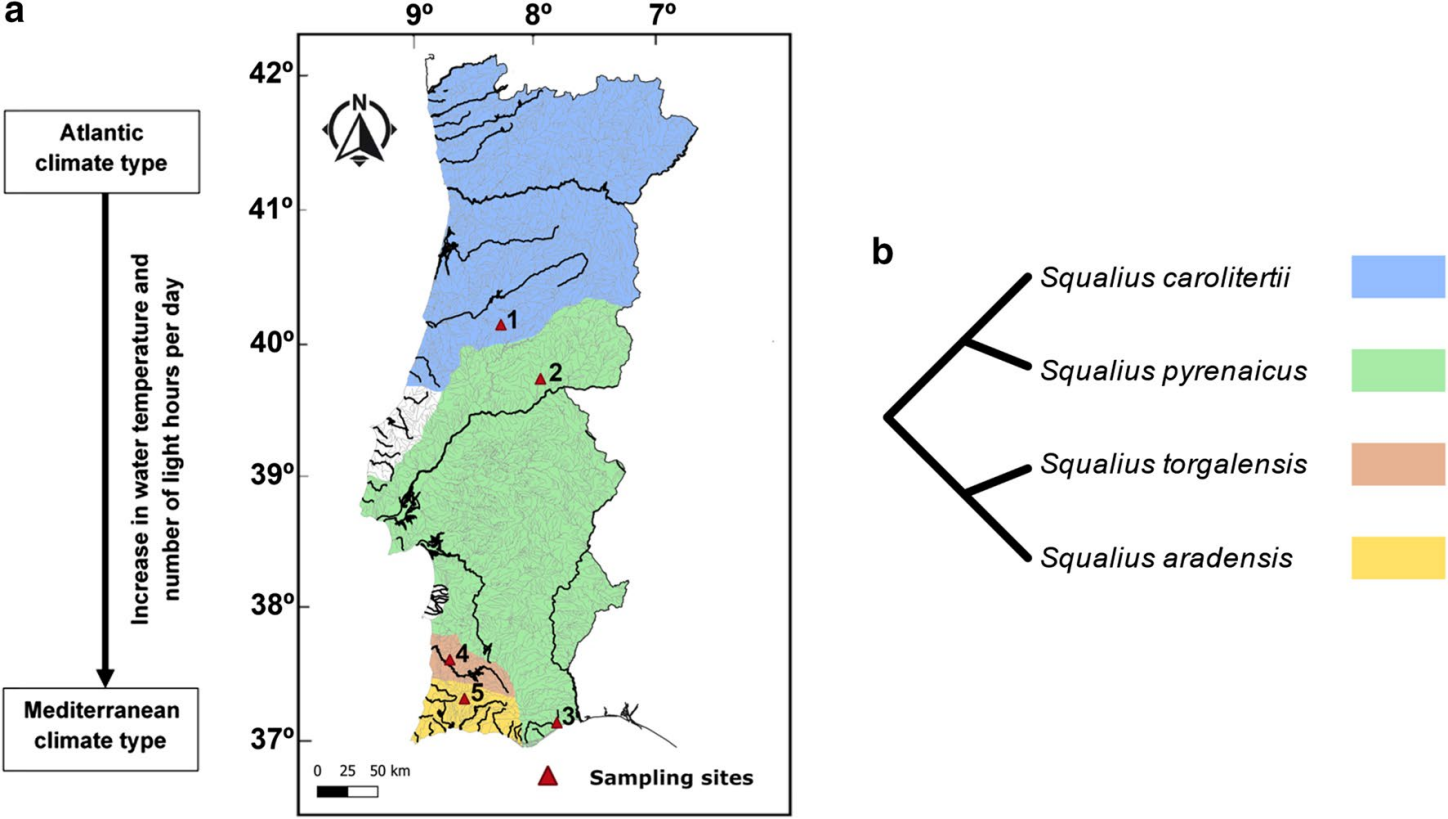
**a** Spatial distribution of the four Western Iberian *Squalius* species in Portugal, sampling sites and representation of environmental differences. **b** Cladogram illustrating the phylogeny of the four Western Iberian *Squalius* species, according to Sousa-Santos et al. (Ref. 28). Sampling sites are marked with red triangles: Mondego basin (1, Sótão river); Tagus basin (2, Ocreza river); Almargem basin (3, Almargem stream); Mira basin (4, Torgal stream); Arade basin (5, Odelouca stream). Axes on map represent latitude and longitude, respectively. The map was created with QGIS software (https://qgis.org) and edited with Inkscape (https://inkscape.org)

In the Western Iberian Peninsula, there are four known species of freshwater fish of the genus *Squalius* Bonaparte, 1837 in Portuguese rivers (*S. carolitertii, S. pyrenaicus, S. torgalensis* and *S. aradensis*, Fig. 1). *S. carolitertii* (Doadrio 1988) inhabits the northern river basins, *S. pyrenaicus* (Günther 1868) occurs in the Central and Southern basins (e.g., Tagus, Guadiana and Almargem), while the sister species *S. torgalensis* and *S. aradensis* (Coelho et al. 1998) are confined to small Southwestern basins (e.g., Mira and Arade, respectively). These four species are diploid with a karyotype of 2n = 50 [26, 27], as in zebrafish. Based on phylogenies of nuclear and mitochondrial markers, the species tree of these species is well known, comprising two main groups (Fig. 1): (i) *S. carolitertii* and *S. pyrenaicus*; and (ii) *S. torgalensis* and *S. aradensis* [28, 29]. These species are classified in the same group as zebrafish (Order Cypriniform, suborder Cyprinoidei; Schönhuth et al. 2018), and hence their evolutionary history is characterized by the same whole genome duplication events [14], likely sharing the same paralogous genes. In Western Iberian Peninsula there is a transition between two contrasting climate types (Fig. 1): the Atlantic in the Northern region that is characterized by mild temperatures (inhabited by *S. carolitertii* and *S. pyrenaicus*), and the Mediterranean in the Southern region (inhabited by *S. pyrenaicus, S. torgalensis* and *S. aradensis*) typified by higher temperatures and droughts during summer periods [30–32]. Thus, species inhabiting the southern basins affected by the Mediterranean climate face harsher conditions. Besides differences in spring average water temperature of approximately 5 ºC along the distribution of these species, there are differences in the average spring photoperiod (approximately 15 minutes) between the northern and southern basins. The environmental differences associated with the distribution of *Squalius* species in Portugal make them a good model to study adaptation to different environmental conditions.

Here, we performed an integrative study on the molecular evolution of circadian system genes in *Squalius* species. We aimed to identify the genes involved in the core circadian system in these species and assess their evolutionary history. We combined several approaches, conducting phylogenetic analysis of the identified genes within each gene family in the *Squalius* species and using predicted protein sequences. Finally, we aimed to detect genes and sites under positive selection and correlate those with predicted functional features of the proteins potentially relevant for the response to environmental differences in light and temperature. These results contribute to a better understanding of the mechanisms of adaptation and response of freshwater fish species to climate change.

## Results

### Evolutionary history of circadian‐related gene families

To identify the genes involved in the central circadian system in *Squalius* species, we compared published transcriptome data of *S. torgalensis* and *S. carolitertii* [33] with light-induced zebrafish transcriptomes [17, 18], as these allow to easily identify genes from the circadian system related with light stimuli. From this comparison we identified sixteen genes in the *Squalius* genus belonging to four main gene families (Cryptochromes, Period, CLOCK and BMAL), involved in the main core of circadian system (Additional file 2: Table S1) and already described for other fish species [7–10]. Identified genes were re-sequenced for *S. torgalensis* and *S. carolitertii* and sequenced *de novo* for *S. pyrenaicus* and *S. aradensis*, and exon identity was confirmed based on sequence alignment with zebrafish sequences (Additional file 3: Table S2).

To test if the proteins encoded by the identified genes potentially retain the function involved in the circadian system, we used the predicted protein sequences to conduct an analysis of protein-protein interactions (PPI). This revealed that all proteins interact with other core circadian proteins (Additional file 4: Table S3). We also found proteins with interactions indirectly related with the core circadian system through circadian-dependent functions (Additional file 4: Table S3).

To assess evolutionary history of gene families we conducted phylogenetic analysis independently for each family based on predicted protein sequences (Additional file 1: Fig. S2). To account for paralogous, we used *Drosophila melanogaster* protein as outgroup and included zebrafish (*Danio rerio*) proteins in the phylogeny (see Additional file 3: Table S2 for accession numbers). As detailed for each family below, all reconstructed phylogenies recovered the paralogous evolutionary relationships previously described for other fish species (Additional file 1: Fig. S2) [7–10]. We found that *Squalius* sequences form monophyletic groups clustering with orthologs from zebrafish for all gene families. Therefore, our phylogenies support the identification of orthologs in each gene family.

The gene trees based on nucleotide sequences can be grouped in three major topologies: (1) congruent with the species tree, with two main clusters: (i) *S. aradensis* and *S. torgalensis*; (ii) *S. pyrenaicus* and *S. carolitertii*; (2) incongruent with species tree and with two main clustering groups: (i) *S. carolitertii* and *S. pyrenaicus* from Tagus; and (ii) *S. torgalensis, S. aradensis* and *S. pyrenaicus* from Almargem; and (3) incongruent with the species tree and with two main clustering groups: (i) *S. carolitertii*, *S. pyrenaicus* from Tagus and S. *torgalensis*; and (ii) *S. aradensis* and *S. pyrenaicus* from Almargem (Table 1, Additional file 1: Fig. S3). These incongruences can be due to incomplete lineage sorting or selection (discussed below). Three additional topologies were recovered for three genes, likely resulting from incomplete lineage sorting (Table 1, Additional file 1: Fig. S3).

**Table 1:** Topology of gene trees and summary of tests for positive selection and increased nonsynonymous rate in specific lineages

Topology of phylogenetic gene tree	Genes	Gene-wide test for positive selection^a^	Branch test for positive selection^b^		Site-level positive selection^c^	Increased nonsynonymous rate^d^	
		Positive selection?	Positive selection?	Branch of the gene tree under selection	Positive selection?^e^	(Sc, SpT, SpG) vs. (St, Sa) clades*^,e^	Convergent lineages^e^
Species tree ((Sc,SpT),SpG),(Sa,St)*	*cry1ba*	✖	✖	**–**	✖(–)	✔ (1)	**–**
	*per1a*	✖	✖	**–**	✔ (6)	✔ (3)	**–**
	*per3*	✔	✔	*S. pyrenaicus (Almargem)*	✔ (6)	✔ (3)	**–**
	*bmal2*	**✔**	✖	**–**	✔ (5)	✔ (11)	**–**
Convergence (Sc,SpT),(St,(Sa,SpG))*	*cry1aa*	✖	✖	**–**	✔ (1)	**–**	✖(–)
	*cry1bb*	✖	✖	**–**	✖(–)	**–**	✔ (11)
	*clockb*	✔	✔	*S. carolitertii*	✔ (3)	**–**	✖(–)
	*clock2*	✖	✖	**–**	✔ (1)	**–**	✔ (3)
Convergence ((Sc,SpT),St),(Sa,SpG)*	*cry3*	✖	✖	**–**	✔ (14)	**–**	✔ (12)
	*per1b*	✔	✔	*S. torgalensis*	✔ (1)	**–**	✖(–)
	*per2*	✔	✔	*S. torgalensis +* *S. aradensis*	✔ (15)	**–**	✔ (1)
	*clocka*	✖	✖	**–**	✔ (2)	**–**	✖(–)
	*bmal1a*	✖	✖	**–**	✖(-)	**–**	✖(–)
Other topologies	*cry1ab*	✖	✖	**–**	✖(-)	**–**	**–**
	*cry2*	✖	✖	**–**	✔ (1)	✔ (2)	**–**
	*bmal1b*	✖	✖	**–**	✖(–)	**–**	**–**

*Sc, *Squalius carolitertii*; SpT, *Squalius pyrenaicus* (Tagus population); SpG, *Squalius pyrenaicus* (Almargem population);

St, *Squalius torgalensis*; Sa, *Squalius aradensis*.

^a^BUSTED[78]; ^b^aBSREL[80, 81]; ^c^MEME[79] and FUBAR[83]; ^d^Contrast-FEL[84]; ^e^Number of sites within parenthesis

### Signatures of selection in circadian‐related genes

**Fig. 2:**
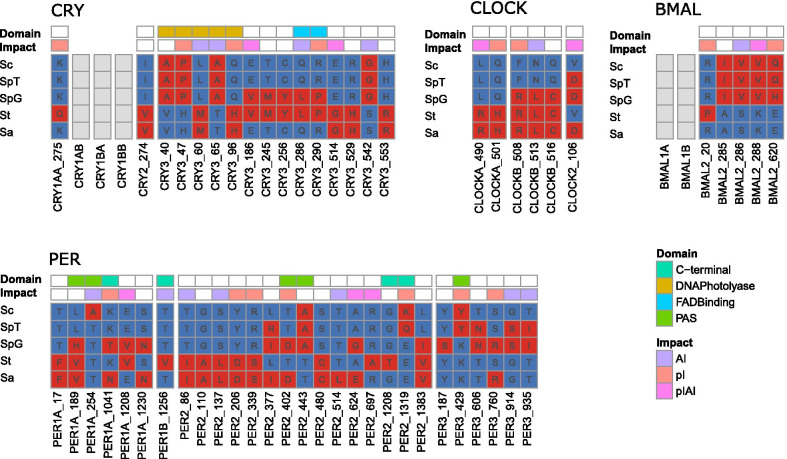
Sites under positive selection detected with MEME and FUBAR. For each gene family the protein, site (coded as protein name_site), corresponding amino acids in the different populations/species, protein domain and predicted impact are shown. For each site, the likely derived amino acid change is shown in red and the ancestral state in blue, obtained by comparing with the outgroup *Danio rerio*. Proteins where no sites under positive selection were detected are shown in gray. Species abbreviations: Sc: *Squalius carolitertii*; SpT: *S. pyrenaicus* (Tagus); SpG: *S. pyrenaicus* (Almargem); St: *S. torgalensis*; Sa: *S. aradensis*. See Additional file 6: Table S5 and Additional file 1: Fig. S4 for further information

**Fig. 3:**
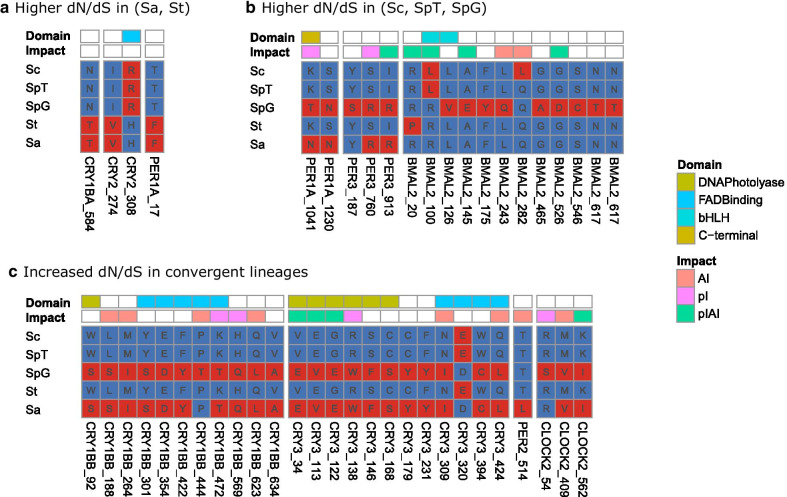
Sites with evidence of increased dN/dS detected with contrast-FEL. **a** Increased dN/dS in (*S. aradensis*, *S. torgalensis*) clade. **b** Increased dN/dS in (*S. carolitertii*, *S. pyrenaicus* Tagus, *S. pyrenaicus* Almargem) clade. **c** Increased dN/dS in (*S. pyrenaicus* Almargem, *S. aradensis*) convergent lineages. For each gene family the protein, site (coded as protein name_site), corresponding amino acids in the different populations/species, protein domain and predicted impact are shown. For each site, the likely derived amino acid change is shown in red and the ancestral state in blue, obtained by comparing with the outgroup *Danio rerio*. Species abbreviations: Sc: *Squalius carolitertii*; SpT: *S. pyrenaicus* (Tagus); SpG: *S. pyrenaicus* (Almargem); St: *S. torgalensis*; Sa: *S. aradensis*. See Additional file 8: Table S7, Additional file 9: Table S8 and Additional file 1: Fig. S4 for further information

Given the confidence on gene identity, we tested for signatures of natural selection with several statistical tests based on dN/dS ratio. Out of 16 genes, we detected positive selection in five using the gene-wide level test implemented in BUSTED (Table 1; Additional file 5: Table S4), and in 11 using the site-level analysis implemented in MEME and FUBAR to detect individual sites under positive selection (Additional file 6: Table S5). Signatures of positive selection were found in 55 sites (Fig. 2, Additional file 1: Fig. S4). Comparing the amino acids in the four *Squalius* species and zebrafish (outgroup) at sites detected to be under positive selection, we found that most amino acid changes were non-conservative (Additional file 6: Table S5) with a predicted impact on either isoelectric point, aliphatic index or both (Fig. 2). Interestingly, for most sites the amino acid changes were in species inhabiting the Mediterranean climate type (31 sites in *S. torgalensis*, 28 in *S. aradensis*, and 31 in *S. pyrenaicus* population from Almargem, Fig. 2). Among the genes with signatures of positive selection for at least one of the mentioned methods, we detected significant positive selection in at least one external branch of the phylogeny in five of them, using a branch-based test implemented in aBSREL (Table 1; Additional file 7: Table S6). Using contrast-FEL we detected 6 sites with evidence of increased dN/dS likely due to stronger positive selection in the clade of *S. aradensis* and *S. torgalensis* in CRY1BA, CRY2 and PER3, which code for genes known to respond to temperature in zebrafish [21, 23]; as well as 3 sites in PER1A, which was shown in previous studies to change gene expression in *S. carolitertii* and *S. torgalensis* exposed to different temperatures [24, 25] (Fig. 3a, Additional file 8: Table S7). Also, 11 sites of BMAL2 showed significantly higher dN/dS in the lineage of *S. pyrenaicus* from Almargem (Fig. 3b, Additional file 8: Table S7).

Furthermore, we found evidence for increased dN/dS in lineages with gene trees consistent with convergence (*S. aradensis* and *S. pyrenaicus* from Almargem) at 11 sites of CRY1BB, 12 sites of CRY3 (mostly on DNA Photolyase and FAD-Binding domains), 1 site of PER2 and 3 sites of CLOCK2 (Fig. 3c, Additional file 9: Table S8). At almost all these sites except in 3 both species from the convergent lineage shared the same amino acid, in most cases a non-conservative change with likely impacts on the aliphatic index and/or isoelectric point of the proteins (Fig. 3c, Additional file 9: Table S8).

Additionally, we tested for purifying selection with the FEL and FUBAR statistical tests to detect sites under negative selection. We found a considerable number of conserved positions under negative selection (282 sites in total), ranging from 3 sites in *cry1ba* to 59 sites in *per2* (Additional file 1: Fig. S5, Additional file 10: Table S9). Moreover, approximately 1/3 of these sites are in codons for aliphatic amino acids (alanine, isoleucine, leucine, proline, and valine) (Additional file 10: Table S9).

### Prediction of functional and structural features of circadian‐related proteins

To investigate possible functional modifications on the proteins related with adaptive changes, we predicted the aliphatic index (AI) and isoelectric point (pI) for these proteins based on the amino acid sequences. These parameters were chosen due to their relationship with protein thermostability, a relevant factor for the studied species, given that they inhabit regions with distinct water temperatures. While AI is defined as the relative volume occupied by aliphatic side chains (Ala, Val, Ile, Leu) and it is positively correlated with increased thermostability of globular proteins [34, 35]; pI reflects the pH at which a protein has neutral net charge, which is indicative of the overall composition of charged amino acids, and hence it is important for protein subcellular localization and potential electrostatic interactions responsible from protein stabilization [35–37].

Regarding the predicted aliphatic index (AI), we found differences among *Squalius* species for all proteins except for BMAL1B. For CRY1AA, CRY1AB and PER1A we found that northern populations of *S. carolitertii* and *S. pyrenaicus* from Tagus presented higher values of AI, whereas for CRY3, PER2, PER3, CLOCKB and BMAL1A the southern populations showed higher AI (Additional file 11: Table S10). We found differences in the predicted pI across *Squalius* species for most proteins, except for PER1B, CLOCKA, BMAL1A, BMAL1B (Additional file 11: Table S10). The pI values tended to be lower in most proteins of southern populations of *S. torgalensis, S. aradensis* and *S. pyrenaicus* from Almargem, even though this was not the case for CRY1BA, CRY1BB, CRY3, PER3, CLOCKB, CLOCK2 and BMAL2 (Additional file 11: Table S10).

We also conducted a sequence-based analysis to predict domain and/or motif location using HMM-based methods (Additional file 1: Figs. S4, S5). For CRY proteins, we found two common domains to all CRY proteins: the DNA photolyase domain and the FAD-binding domain. Both domains are responsible for binding chromophores and may be of extreme importance for the activity of these proteins [10]. We found two domains common to all PER proteins: Period-Arnt-Sim (PAS_3/PAS_11) domain and the Period protein 2/3 C-terminal region. Additionally, PER1A, PER2 and PER3 present an additional PAS domain relatively well conserved. PAS domains are of extreme importance for PER function as they serve for protein dimerization, but also display activity of photoreception [38, 39]. For CLOCK and BMAL proteins, we found three domains conserved in all two proteins: two Period-Arnt-Sim (PAS fold and PAS_11) domain and the basic helix-loop-helix (bHLH). The bHLH is a protein structural motif that characterizes one of the largest families of dimerizing transcription factors and consists in a DNA-binding region.

## Discussion

The circadian system generates and maintains endogenous rhythms synchronised with the daily fluctuation of light and is observed in a wide range of life forms [2, 3]. We identified and characterized the molecular evolution of genes from the core circadian system in western Iberian chubs, freshwater fish species of genus *Squalius* that inhabit different river basins under two climatic types (Atlantic and Mediterranean), with differences in light and temperature.

### Identification of paralogs and orthologs of all circadian gene families in *Squalius*

We were able to identify sixteen genes (Additional file 2: Table S1) belonging to the four known core-circadian gene families (CRY, PER, CLOCK and BMAL), which are orthologous to genes described in *D. rerio* and other fish species [6–10].

Phylogenetic relationships within each gene family revealed that the history of paralogs genes is conserved in *Squalius* species, and supports the correct identification of circadian orthologs and paralogs [6]. Moreover, in agreement with results from *Danio rerio* and other fish species [21, 22, 40, 41] (summary on Additional file 2: Table S1), we detected possible diversification in functions of the circadian genes that may have arisen to optimize important biological processes, synchronised with the circadian oscillation. We found that in *Squalius* the set of predicted protein-protein interactions in CRY, PER and BMAL (Additional file 4: Table S3) involve circadian related proteins, but also proteins with other biological functions, namely BHLHe41 (basic helix-loop-helix family, member e41) and HSF2 (heat-shock factor 2), involved in temperature response [23, 42–44], or with NFIL3-5 (nuclear factor, interleukin 3-regulates, member 5), a protein involved in immune response [45].

### Evidence of positive selection is related with protein predicted function

We found evidence of positive selection mostly on *cry* (*cry1aa*, *cry2* and *cry3*) and *per* (*per1a, per1b, per2* and *per3*) genes (Table 1 and Additional file 5: S4, Additional file 6: Table S5, Additional file 7: Table S6). These genes encode the negative elements of the circadian system acting as repressors of transcription [46] (Additional file 1: Fig. S1). These genes are sensitive to environmental stimuli (e.g. light or temperature) and refine the regulation of the circadian system (e.g. [13, 22, 23]). Moreover, within these genes, we found 44 potential adaptive changes many of which located within the functional domains of the protein, namely in CRY3, PER1A, PER1B, PER2 and PER3 (Fig. 2, Additional file 1: Fig. S4, Additional file 6: Table S5). In CRY3 many changes were located inside the DNA Photolyase domain, one of the light-sensitive domains of cryptochromes. In PER proteins, some adaptive substitutions were located inside PAS domains (Fig. 2, Additional file 1: Fig. S4) that serve for dimerization with CRY proteins [38, 39]. Several changes in PER2 and in PER3 are non-conservative that alter the charge of the amino acid, which can consequently alter the strength of protein-protein interactions. Moreover, these changes can impact protein structure as certain amino acids have a propensity for specific structural arrangements [47]. Although there are more *cry* and *per* genes (10) from the negative loop than *clock* and *bmal* genes (6) from the positive loop, the proportion of sites under positive selection in the negative loop genes is larger than expected if sites were randomly distributed (Additional file 12: Table S11). A possible explanation is that genes of the positive loop are under stronger selective constraints due to purifying selection, however this is not fully supported by our results that indicate more sites under negative selection in *cry* and *per* genes (Additional file 8: Table S9). Another possibility is that due to a higher number of paralogous genes in the negative loop these could have evolved to respond to different environmental stimuli, which is supported by our results on predicted protein interactions (Additional file 4: Table S3). The fact that we find positive selection in the negative elements is in line with previous studies showing that *cry* and *per* genes are important to integrate stimuli other than light in fish (e.g. mutations in PER2 protein were important for the adaptation of blind cavefish to its environment [48]). Taken together, these results suggest that circadian system genes were involved in Iberian *Squalius* species adaptation, which occurred mostly by changes in the negative elements of the circadian system.

### More genes under positive selection in populations under mediterranean climate

Based on dN/dS we found evidence of positive selection in all species using different tests (Table 1, Additional file 5: Table S4, Additional file 6: Table S5, Additional file 7: Table S6). The site level tests indicate that signatures of positive selection are present mostly in southern populations (*S. torgalensis, S. aradensis* and *S. pyrenaicus* from Almargem, Fig. 2 and Additional file 6: Table S5) that are under the influence of the Mediterranean climate type. These results indicate that circadian genes can be involved in adaptation of these species to their specific environments. The circadian system is indirectly related to regulation of many physiological and metabolic aspects that affect the response of organisms to environmental stimuli, and hence these signatures of positive selection can be related to adaptation due to several environmental factors. Despite light stimuli, temperature is important for the proper maintenance of the circadian system in fish [21–23], and temperature is known to impose strong selective pressures in ectothermic species [49, 50]. Thus, present-day and past differences in temperature between river drainage systems inhabited by *Squalius* species can explain our results.

There are several lines of evidence supporting the hypothesis that temperature is a key selective pressure. First, populations with more genes and sites with signatures of positive selection inhabit a region influenced by a Mediterranean climate with higher water temperatures (Fig. 2, Additional file 6: Table S5). Second, evidence from protein analysis shows differences in predicted protein thermostability between species in different climatic types (Additional file 11: Table S10). A higher protein thermostability can be achieved either by (i) increasing the aliphatic index AI [34, 35], (ii) increasing the strength of ionic interactions [51], or a combination of these mechanisms. We found that in 55 putatively adaptive changes of the core circadian genes, 13 of them (in CRY3, PER1A, PER1B, PER2, PER3, BMAL2, and CLOCKB) have a potential impact on protein aliphatic index (AI), 15 of them (in CRY1AA, CRY3, PER1A, PER2, PER3, BMAL2, CLOCKA, CLOCKB) on isoelectric point (pI), and 8 of them in both AI or PI (in CRY3, PER1A, PER2, BMAL2, CLOCK2 and CLOCKA), and therefore can have a direct effect on protein thermostability (Additional file 6: Table S5). For these proteins, the analysis of predicted AI and pI showed differences between the *Squalius* species inhabiting the Atlantic and Mediterranean climate types (Additional file 11: Table S10), suggesting that differences in protein thermostability can result from adaptation to different water temperatures. Moreover, approximately one third of the sites inferred to be under negative selection were on codons encoding for aliphatic amino acids (Additional file 10: Table S9), which are associated with protein thermostability.

Last, signatures of positive selection were found mostly on *cry* and *per* genes, circadian genes that have been shown to regulate temperature integration within the circadian system in fish (Additional file 2: Table S1) [21, 22, 24, 25]. For instance, we found signatures of positive selection and/or differences in adaptive substitution rate in lineages from different environments (Table 1) in *cry1ba, cry2*, *per1b* and *per3*, which are four genes known for integrating temperature within the circadian system in *D. rerio* (Additional file 2: Table S1) (see also [21]). For instance, for PER3 protein we detected positive selection at site K429Y in the PAS domain, with an amino acid change in *S. carolitertii* and *S. pyrenaicus* from Tagus predicted to affect the isoelectric point (Fig. 2, Additional file 6: Table S5), reflected in differences in pI between *Squalius* from northern and southern river basins (Additional file 11: Table S10). Another clear example is *per1b*, for which *S. torgalensis* has the PER1B with the highest AI and pI (Additional file 11: Table S10) and we found signatures of positive selection in *S. torgalensis* at a site associated with amino changes that increase AI (Fig. 2, Additional file 6: Table S5), suggesting that selection led to increased thermostability. This protein has been shown to be important in *D. rerio* for the integration of temperature and light cues within the circadian system [21]. We also found signatures of positive selection in *cry1aa* and *per1a*, both found to change their gene expression in *S. torgalensis* and *S. carolitertii* when exposed to increased water temperature under controlled laboratory conditions [24, 25]. In PER1A, we found a mutation under positive selection in *S. carolitertii* at an important region of the protein (PAS domain, Fig. 2, Additional file 1: Fig. S4, Additional file 6: Table S5) that leads to an increase in the aliphatic index, and therefore increased protein thermostability. This is compatible with Jesus et al. 2017 [25], that observed a downregulation of the expression of this gene at higher temperatures in *S. carolitertii*. We speculate that such increase in protein thermostability could have been selected to properly function at higher temperatures with low expression levels, compensating the costs of over-expression of other proteins.

### Evidence of adaptive convergence

In four genes (*cry1ba, per1a, per3* and *bmal2*) the best topology for the inferred gene tree is congruent with the species tree with two main clusters (Fig. 1; Table 1, [28, 29]). This could indicate that these genes evolved neutrally during speciation. However, we found signatures of positive selection in three of them (*per1a, per3* and *bmal2*) and differences in dN/dS ratio between the two clades in the four genes (Fig. 3a, b, Additional file 8: Table S7), which might indicate that the evolution of these genes has been at least partially driven by natural selection, rather than exclusively by neutral divergence. However, the phylogenies we inferred using both the nucleotide and protein sequences indicate that for 9 out of 16 genes (*cry1aa, cry1bb, cry3, per1b, per2, clockb, clock2* and *bmal1a*) the best topology clusters together, with high support, *S. aradensis* and *S. pyrenaicus* from Almargem (Additional file 1: Fig. S2 and S3). According to the species tree these two species belong to two highly divergent lineages (Fig. 1) [28], and hence such a high proportion of genes with this clustering is unlikely due to neutral incomplete lineage sorting. Instead, this suggests a scenario of evolutionary convergence of populations inhabiting similar environments. In fact, Almargem and Arade are two basins from the south of Portugal influenced by Mediterranean climate, facing very similar environmental conditions (e.g., average water temperature and photoperiod). This pattern of sequence convergence in these genes and proteins may thus be a consequence of convergent adaptation.

The convergence in these genes and proteins matches at least partially the criteria for detecting evolutionary convergence proposed by Dávalos et al. (2012), namely: (1) evidence from sequences of functional parts of genes; (2) clear link between gene function and ecological conditions; and (3) evidence that selection is acting on target genes at different rates from other lineages. As we only obtained data from cDNA, we expect all the sequences to be from exons, and therefore, constituting functional parts of genes, confirming the criterium (1). As previously mentioned, most genes presenting this signal of convergence belong to CRY and PER family, and both *cry* and *per* genes have a demonstrated importance in the response to environmental temperature [21], therefore confirming the criterium (2). Based on dN/dS tests we could confirm the criterium 3 for *cry1bb, cry3*, *per2* and *clock2*, since we found increased dN/dS in the lineages with evidence of convergence (Fig. 3c; Table 1, Additional file 9: Table S8). Furthermore, for *per2* and *clockb* we found sites under positive selection with amino acid changes shared by *S. aradensis* and *S. pyrenaicus* from Almargem (Fig. 3c, Additional file 9: Table S8). For *cry1aa, per1b* and *clockb*, protein analysis supports a strong similarity between the physicochemical parameters estimated for *S. aradensis* and *S. pyrenaicus* from Almargem, pointing to a functional convergence at protein level (Additional file 11: Table S10), even though for those genes signatures of positive selection were mostly on *S. torgalensis*. For *bmal1a* we did not find signatures of positive selection or increased dN/dS in convergent lineages, but the functional characterization of BMAL1A protein revealed similar predicted protein physicochemical patterns in *S. aradensis* and *S. pyrenaicus* from Almargem, pointing to functional convergence.

Scenarios of convergence have been described for other species at the morphological level [53–55], and at the molecular level [56–61]. Moreover, light was shown to be an important determinant in two studies that detected molecular convergence in fish, such as in: (1) the evolution of albinism linked to the *Oca2* gene in two independent populations of the cavefish *Astyanax mexicanus* [56]; and (2) in functional evolution of Rhodopsin proteins in several fish species [62]. Here, we detected adaptive convergent evolution in freshwater fish in genes and proteins related to integration of visual and thermal stimuli within the circadian system, which are part of gene families with duplications. This raises the possibility that gene duplications can be important in convergent evolution, which could be further studied and tested in the future. Sequences from genomic DNA could be particularly informative and increase the power to investigate patterns of adaptive convergence, for instance by comparing patterns of genetic divergence at exons and introns.

## Conclusions

This study aimed to characterise the evolution of circadian-related gene families in non-model freshwater organisms. These results provide clear insights on how the environment can shape the evolution of the circadian system, and how it can contribute to the process of adaptation to different environments.

Our findings support that together with neutral historical factors, natural selection also drove the molecular evolution of the four studied species that live in different environmental conditions of light and temperature, affected by Atlantic and Mediterranean climate types. Moreover, we find evidence for adaptive convergence between the two southern populations of *S. aradensis* and *S. pyrenaicus* from Almargem, a pattern described for the first time. Our results using an approach combining dN/dS and protein analysis can help understanding the genetic patterns found in other species, namely those that are due to convergence, a process that can be frequent but difficult to detect.

## Methods

### Sampling

Muscle tissue from five wild adult fish of *S. carolitertii* and *S. torgalensis* species was available from previous work [25] and stored at − 80 ºC in RNALater® (Ambion, Austin, TX, USA). The adult fish were sampled in Portuguese basins of Mondego (40° 8′ 5.22" N; 8° 8′ 35.06" W) and Mira (37° 38′ 1.31" N; 8° 37′ 22.37" W), respectively [25]. Samples of *S. pyrenaicus* were also stored at -80ºC from previous projects. This work includes two sampling sites: Almargem (37°09′ 50.7" N; 7° 37′ 13.2" W) [63] and Tagus (39° 43′ 48.2" N; 7° 45′ 38.1" W) [64].

*Squalius aradensis* individuals were captured from Portuguese basin Arade (37º17′ 0.53’’N; 8º29′ 7.31’’W) under the license 421/2017/CAPT issued by Portuguese authority for Conservation of endangered species [ICNF (Instituto da Conservação da Natureza e das Florestas)]. After capturing, the specimens were transported alive to the laboratory in aerated containers to minimize fish discomfort and euthanized immediately upon arrival with an overdose of tricaine mesylate (400 ppm of MS-222; Sigma-Aldrich, St. Louis, MO, USA) with sodium bicarbonate (1:2) following the recommended ethical guidelines (ASAB/ABS, 2012) and European Union regulations. Organs were stored in RNALater® at -80ºC until further use. Distribution of the species and sampling sites are illustrated in Fig. 1.

### Identification of predicted circadian system related genes in Iberian freshwater fish based on transcriptome analysis

Previously published transcriptome assemblies of *S. torgalensis* and *S. carolitertii* obtained from RNA-Seq experiments [33], were used to identify the genes related to the circadian system in the study species. To identify potential genes related to the circadian system we performed BLAST searches of the transcriptomes of the two species against two *Danio rerio* light-induced transcriptomes [17, 18]. To account for splicing isoforms and to avoid the misidentification of potential paralogous genes, we used a stringent e-value threshold of 1 × 10^− 7^ for the BLAST searches and kept only sequences with identity higher than 85%. To predict the biological and molecular function of the identified genes, we performed a functional enrichment analysis using the list of top blast *Danio rerio* ENA accession numbers (Additional file 3: Table S2) and the method implemented in DAVID functional annotation tool. Through this methodology we were able to find enriched GO terms among the genes retrieved. The most significant enriched GO terms for Biological Process and Molecular Function were filtered, and only the core genes from circadian system (i.e., those described to be involved in the feedback loop) were further kept (Additional file 2: Table S1). A threshold for adjusted p-values (Benjamini) of 0.05 was used to remove false positives.

### Gene sequencing and protein sequence prediction

Based on the sequences retrieved from the transcriptomes of *S. torgalensis* and *S. carolitertii* that matched core genes of the circadian system, we designed specific primers for Polymerase chain reactions (PCRs) using PerlPrimer software v.1.1.19 [65] (Additional file 13: Table S12) with the purpose of amplifying the coding regions of those same genes for all the studied species. We included *D. rerio* sequences (Additional file 3: Table S2) during primer design to identify conserved regions among Cyprinoidae. To further distinguish orthologs from paralogs we performed phylogenetic analysis (see below).

Total RNA was extracted from muscle samples of 25 individuals, 5 from each population. RNA was used to facilitate gene sequencing since introns are avoided. Only muscle tissue was used to avoid tissue expression bias. We added 1 mL TRI Reagent (Ambion, Austin, TX, USA) to 50–100 mg of muscle samples and, after homogenization with Tissue Ruptor (Qiagen, Valencia, CA, USA), extracted RNA according to the TRI Reagent manufacturers protocol. TURBO DNase (Ambion, Austin, TX, USA) was employed to degrade any remaining genomic DNA contaminants, followed by phenol/chloroform purification and LiCl precipitation [66]. Sample quality was checked using a NanoDrop™-1000 spectrophotometer (Thermo Fisher Scientific, Waltham, MA, USA) based on the 260 nm/280 nm and 260 nm/230 nm absorbance ratios. Samples concentration was determined with Qubit® 2.0 Fluorometer (Thermo Fisher Scientific, Waltham, MA, USA) to ensure enough quantity of homogeneous RNA for cDNA synthesis. Synthesis of cDNA was performed according to manufacturer's protocol using the RevertAid H Minus First Strand cDNA synthesis kit (Thermo Fisher Scientific, Waltham, MA, USA), and it was stored subsequently at −20 °C until further use. PCRs were performed in 25 µL reactions containing 10–100 ng of cDNA, 2 mM MgCl_2_, 2 mM each dNTP, 10 µM each primer, Taq Polymerase (5 U/µL), and 1× Taq buffer using thermocycler conditions described in Additional file 14: Table S13. PCR products were confirmed using a 1% agarose gel electrophoresis, and after purification with ExoSAP-IT® PCR Product Cleanup (Affymetrix, Inc., Santa Clara, CA, USA), they were sequenced by Sanger sequencing.

Sequences were aligned and edited using Sequencher v.4.2 (Gene Codes Corp., Ann Arbor, MI, USA). Non-redundant nucleotide sequences were deposited in European Nucleotide Archive (ENA) database under the accession numbers available in Additional file 2: Table S1. CLC Sequence Viewer v.7.5. (CLC bio, Aarhus, Denmark) was used to predict protein sequences for *in silico* analysis. BLAST searches were conducted with resulting protein sequences against UniProt database [67] to ensure their identity. Protein sequences for *Danio rerio* were retrieved from UniProt database for each protein (Additional file 3: Table S2), as well as *Drosophila melanogaster* homolog sequence for each gene family (Additional file 3: Table S2) to use as outgroup in phylogenetic analysis (see below). Protein sequences were aligned by gene family using the M-Coffee method, that combines several alignment algorithms (e.g., MUSCLE, MAFFT and CLUSTAL) [68] available in the T-Coffee web server [69]. For each individual gene, nucleotide sequences of *Squalius* species were also aligned using M-Coffee.

### Phylogenetic analysis and molecular evolution

The most appropriate model for amino acid substitution for each data set was determined with ProtTest v.3.0 [70], using both the Akaike information criteria and Bayesian information criteria. Phylogenetic trees were reconstructed for each gene family independently using the protein sequences and the Bayesian Inference method implemented in MrBayes v.3.2.6 [71, 72], using *D. melanogaster* protein sequences (Additional file 3: Table S2) as outgroup. The Monte Carlo Markov Chain (MCMC) were ran for 500,000 iterations using the parameters determined in ProtTest for protein sequences as priors. Trees were sampled every 500 iterations during the analysis. The first 50,000 iterations were excluded as burn-in after examining the variation in log-likelihood scores over time. Phylogenetic trees were constructed using protein sequences instead of nucleotide sequences to avoid bias from the third codon rapid evolution. Protein sequences used correspond to the direct translation of nucleotide sequences under the standard genetic code. These phylogenetic trees allowed to confirm the correct identification of orthologs and paralogs.

Gene trees were reconstructed for each gene based on nucleotide sequences using a Maximum Likelihood approach on RAxML v.8 [73] only including *Squalius* sequences. The most appropriate model for nucleotide substitution was determined using MEGA X [74].

All trees were edited in FigTree v1.4.2 (A. Rambaut, University of Edinburgh, UK; http://tree.bio.ed.ac.uk/software/figtree/).

### Analysis of signatures of selection based on dN/dS

Signatures of selection were examined based on the dN/dS ratio (also known as the parameter ω) using six methods implemented in HyPhy [75] through the Datamonkey adaptive evolution webserver (Kosakovsky Pond & Frost 2005; Weaver et al. 2018 ; http://www.datamonkey.org/; accessed in August 2018 and October 2020), including (1) BUSTED (Branch-site Unrestricted Statistical Test for Episodic Diversification) [78] that provides a gene-wide test for positive selection, i.e. it estimates one ω per gene; (2) MEME (Mixed Effects Model of Evolution) [79] a mixed-effects maximum likelihood approach to test the hypothesis that individual sites have been subject to positive selection, i.e. estimating variable ω among sites; (3) aBSREL (adaptive Branch-Site Random Effects Likelihood) [80, 81] for genes whose signals of positive selection were detected with BUSTED or MEME, to test for positive selection on particular branches of the gene tree, i.e. estimating different ω for different branches; (4) FEL (Fixed Effects Likelihood) [82], which uses a maximum likelihood approach to infer nonsynonymous (dN) and synonymous (dS) substitution rates on a per-site basis for a given coding alignment and corresponding phylogeny to test the hypothesis that individual sites have been subject to negative selection; (5) FUBAR (Fast, Unconstrained Bayesian AppRoximation) [83], which uses a Bayesian approach to infer the posterior probabilities that nonsynonymous (β) substitution rates is higher (indicating positive selection β > α) or lower (indicating negative selection α > β) than the synonymous (α) substitution rate for each site; and (6) Contrast-FEL (fixed effects site-level model) [84] that uses a likelihood ratio test to compare the nonsynonymous substitution rate of different sets of branches. For aBSREL we estimated different  ω= dN/dS for each external branch of the inferred gene trees (i.e., the phylogenetic tree of each gene). We used Contrast-FEL to test for differences in the nonsynonymous rates in different clades. For genes with an inferred gene tree with the same topology as the species tree (*cry1ab*, *per1a*, *per3*, *bmal2*) we tested for differences between the two major clades (*S. torgalensis*, *S. aradensis*) and (*S. carolitertii*, *S. pyrenaicus* Tagus, *S. pyrenaicus* Almargem), specifying as the foreground either the external and ancestral branches or just the ancestral branch of *S. torgalensis* and *S. aradensis* clade. This was also done for the gene *cry2* since it had a well defined (*S. carolitertii*, *S. pyrenaicus* Tagus, *S. pyrenaicus* Almargem) clade that was treated as the background. For genes with inferred genes trees consistent with convergence (*cry1aa*, *cry1bb*, *cry3*, *per1b*, *per2*, *clocka*, *clockb*, *clock2*, *bmal1*) we further tested for differences between the convergent (*S. aradensis*, *S. pyrenaicus* Almargem) clade and the other branches, specifying as the foreground either the external and ancestral branches or just the ancestral branches of *S. aradensis* and *S. pyrenaicus* Almargem clade. For all methods we used the default settings. For all maximum likelihood methods we considered tests with a *p*-value < 0.1 as statistically significant, whereas for the Bayesian approach implemented in FUBAR we considered a posterior probability larger than 0.90 (corresponding to a Bayes Factor > 9.0). For the sites potentially under positive selection detected with MEME, FUBAR and Contrast-FEL we compared the amino acid of the *Squalius* species with the outgroup *Danio rerio*, which was considered the ancestral state. For sites with more than two amino acids where the outgroup was different from any *Squalius*, we considered as the ancestral state the more common amino acid among the *Squalius* species.

### Prediction of functional and structural features of the predicted proteins

Homology methods available on several resources at the ExPASy Server [35] were used to infer several properties of the predicted proteins from cDNA sequences. Specifically, physicochemical parameters of the proteins were predicted using ProtParam [35]. Given the biological significance of factors related to temperature and pH, we predicted the aliphatic index (AI) and the isoelectric point (pI). We tested for differences in physicochemical parameters using several statistical tests implemented in R v.3.2.3 (R Core Team 2015). First, we checked for normality using the Shapiro–Wilk Test. Due to lack of normality, we used a Kruskal-Wallis Rank Sum Test to identify overall statistical differences in parameters across the populations. When evidence for differences were found, we conducted pairwise Wilcoxon Rank Sum Tests to compare the different groups.

To assess further functional features, a sequence-based prediction of family assignment and sequence domains based on collections of Hidden-Markov Models to support the predictions were accomplished for each protein separately using HMMER web server [85–87] against Pfam database [88]. Representative images of structural organization of domains and locations of sites under selection were created and edited with IBS, Illustrator for Biological Sequences [89].

## Supplementary Information

**Additional file 1.** Additional figures.

**Additional file 2: Table S1.** Circadian related genes identified with respective annotations obtained in functional annotation analysis. ENA accession numbers are for non-redundant *Squalius* sequences obtained by Sanger sequencing in this work.

**Additional file 3: Table S2.** List of *Danio rerio* and *Drosophila melanogaster* Uniprot accession ID for target proteins and ENA accession IDs for corresponding coding genes.

**Additional file 4: Table S3.** Patterns of protein-protein interactions for circadian-related protein predicted with STRING v10.5 with a threshold of 0.7 for score.

**Additional file 5: Table S4.** Summary of gene-wide positive selection analysis in circadian-related genes using the BUSTED method implemented in Datamonkey webserver.

**Additional file 6: Table S5.** Summary of sites detected to be under positive selection using MEME and FUBAR methods, and corresponding changes in amino acid and predicted impacts.

**Additional file 7: Table S6.** Summary of branch-site positive selection analysis using the aBSREL method implemented in Datamonkey webserver.

**Additional file 8: Table S7.** Summary of sites with evidence of increased dN/dS ratios (estimated β) on* Squalius torgalensis* and* S. aradensis* clade (foreground) or in* S. carolitertii*,* S. pyrenaicus* clade (background), corresponding changes in amino acids and predicted protein impacts.

**Additional file 9: Table S8.** Summary of sites with evidence of increased dN/dS ratios (estimated β) on lineages of* Squalius pyrenaicus* (Almargem) and* S. aradensis* lineages (foreground) with evidence of convergence, corresponding changes in amino acids and predicted protein impacts.

**Additional file 10: Table S9.** Summary of the results obtained by FEL analysis for pervasive negative selection and with FUBAR for detecting negative selection in coding genes for circadian-related proteins.

**Additional file 11: Table S10.** Predicted physicochemical parameters (AI – Aliphatic index and pI – isolectric point) for each predicted protein.

**Additional file 12: Table S11.** Number of sites in coding proteins for genes of the negative loop (*cry* and *per*) and of the positive loop (*bmal* and *clock*) of circadian-related genes.

**Additional file 13: Table S12.** List of primer pairs and respective sequences used in PCR to (re)sequence circadian-related genes with Sanger method in *Squalius* species.

**Additional file 14: Table S13.** PCR conditions for each pair of primers (Additional file 13: Table S12) used in amplification of circadian-related genes.

## Data Availability

All sequences obtained during the execution of this work are available on the European Nucleotide Archive (ENA) under the accession numbers reported in Additional file 2: Table S1.
